# Standardization of the Umbilical Scarectomy and Exploratory Laparotomy for Umbilical Mucosal Polyps

**DOI:** 10.7759/cureus.71275

**Published:** 2024-10-11

**Authors:** Hirofumi Tomita, Naoki Shimojima, Kentaro Matsuoka, Akihiro Shimotakahara, Miki Ishikawa, Teizaburo Mori, Kiyotomo Abe, Ayano Tsukizaki, Kazuki Hirohara, Seiichi Hirobe

**Affiliations:** 1 Department of Surgery, Tokyo Metropolitan Children’s Medical Center, Tokyo, JPN; 2 Division of Pediatric Surgery, Department of Pediatric Surgical Specialties, National Center for Child Health and Development, Tokyo, JPN; 3 Department of Pathology, Tokyo Metropolitan Children’s Medical Center, Tokyo, JPN

**Keywords:** children, omphalomesenteric band, omphalomesenteric duct remnant, surgical technique, umbilical cyst, umbilical granulation, umbilical sinus

## Abstract

Background

Umbilical mucosal polyps are common, but physicians’ unfamiliarity with them can prolong the patient’s illness. Furthermore, the details of surgery for umbilical mucosal polyp removal are not well documented.

Methods

Patients with an umbilical mucosal polyp diagnosed on the basis of the lesion’s characteristic appearance were prospectively enrolled. The surgery involved an umbilical scarectomy with the removal of a minimum of the surrounding skin and an exploratory laparotomy to detect any lesions extending into the peritoneal cavity.

Results

Fourteen patients with a median duration of illness of ten months (range: one month to seven years) were enrolled, and 13 (92.9%) received surgery while one patient whose symptoms resolved following topical steroid treatment did not. Inspection of the intestinal mucosa of all the patients found lesions deep within the umbilicus in four (30.8%) of the 13 surgical patients, including an omphalomesenteric band, umbilical cyst, and umbilical sinus with gastric mucosa in one, one, and two patients, respectively. The postoperative course was uneventful except for one patient who had temporary granulation.

Conclusions

Umbilical mucosal polyps can be readily diagnosed by their characteristic appearance, thereby preventing the prolongation of illness. An umbilical scarectomy and abdominal exploration may be useful for preventing recurrences and intestinal obstruction.

## Introduction

Umbilical mucosal polyps, which originate in omphalomesenteric duct remnants and are characterized by having a smooth surface and bright-red appearance, are common but poorly recognized even by pediatricians. As a result, they are often misdiagnosed as umbilical granuloma [1], the commonest type of umbilical lesion in neonates and infants, which can be treated non-surgically using silver nitrate cautery, ligation, or topical steroid therapy. By contrast, an umbilical mucosal polyp requires surgical excision for symptom alleviation [1]. Therefore, misdiagnosing an umbilical mucosal polyp as a granuloma can result in the application of ineffective or inappropriate treatments that only prolong the illness.

Some patients with an umbilical mucosal polyp may have other lesions originating from an omphalomesenteric duct remnant [2-4]. However, the prevalence of these lesions and the details of the surgical treatment are not well documented. The tissue beneath the umbilicus contains a cicatricial, retracted umbilical cord remnant [5], which is sometimes referred to anatomically as an “umbilical scar” [6]. The term may also be used for the umbilicus itself [7] or a surgical wound in the umbilicus [8]. Matsukawa et al. recommended the total resection of the anatomical umbilical scar during surgery for an umbilical mucosal polyp because the lesion can extend into or be continuous with the scar tissue [1]. We herein attempted to standardize the total resection technique for anatomical umbilical scars. Borrowing the term “scarectomy,” meaning “scar removal,” from Elias’s study describing the removal of postoperative scars [9], we have coined the term “umbilical scarectomy” to describe anatomical umbilical scar removal rather than postoperative umbilical scar removal. Also included in our discussion is a description of our monocentric experience of using the umbilical scarectomy and exploratory laparotomy to treat umbilical mucosal polyps.

## Materials and methods

Patients and ethical considerations

This study was conducted in Tokyo Metropolitan Children’s Medical Center, Tokyo, Japan. Among the patients who presented to the study center with an umbilical abnormality between April 2018 and March 2023, those with a diagnosis of umbilical mucosal polyp based on their clinical history and the characteristic appearance of the lesion were prospectively enrolled. Patients with different clinical diagnoses, such as umbilical granulation or urachal sinus, were excluded. In the differential diagnosis of reddish, umbilical masses in infants, features indicative of an umbilical mucosal polyp are a smooth surface, bright-red coloration, ineffectiveness of non-surgical interventions, and, occasionally, dermatitis of the surrounding skin caused by digestive discharge. Features indicative of an umbilical granuloma are a rough surface, dull reddish or pink coloration, and resolution within a few months of birth with or without non-surgical intervention. An umbilical mucosal polyp is diagnosed definitively on the basis of histopathological findings in surgical specimens of the intestinal mucosa [1,3]. In patients with mild symptoms, surgery was scheduled for after one year of age, and non-surgical treatments were occasionally administered during the waiting period. In patients with troublesome symptoms, surgery was scheduled immediately.

The patient characteristics, non-surgical treatments, surgical and histopathological findings, and postoperative course were summarized. Categorical data were expressed as a number and percentage, and continuous data were expressed as the median and range. The present study was approved by the Ethics Committee of Tokyo Metropolitan Children’s Medical Center (H29b-126), and the patients’ guardians consented to participate in this study.

Surgical technique

**Figure 1:**
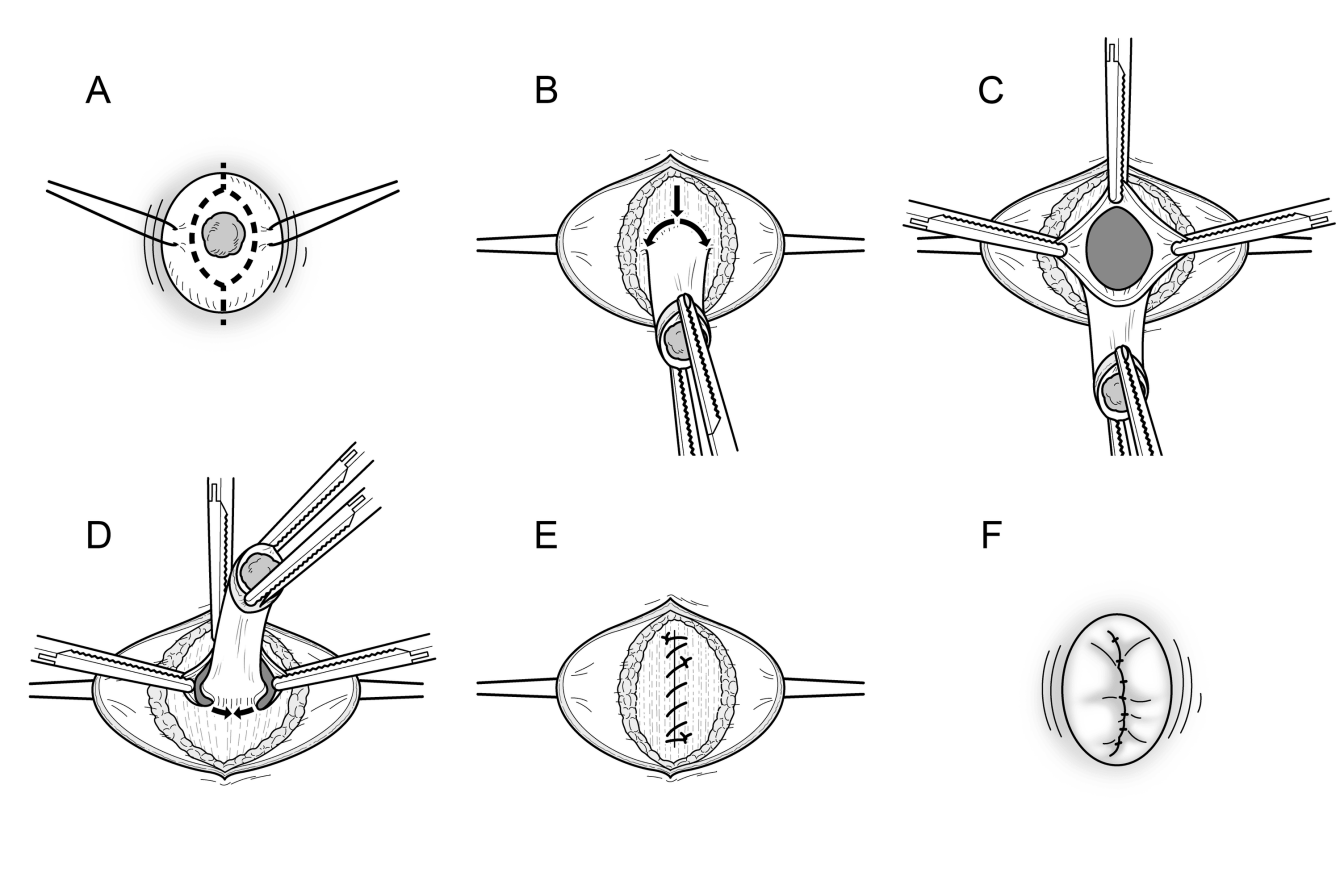
Schema showing the umbilical scarectomy and exploratory laparotomy for umbilical mucosal polyp removal A: A vertical, spindle-shaped incision was made around the umbilical mucosal polyp. B: A vertical incision was made cranially in the linea alba toward the umbilical scar, then extended caudally around the umbilical scar. C: The peritoneum was incised, and the abdominal cavity was explored for any continuous lesions behind the umbilical scar. D: The umbilical scar in each case was totally resected along with the dorsal peritoneum after making a caudal incision between the scar and the linea alba. E: The fascia was closed longitudinally with 2-0 multifilament continuous absorbable sutures. F: The halves of the skin flaps were longitudinally sutured together using dermal 5-0 absorbable multifilament sutures while anchoring the bottom of the umbilicus to the fascia. Image Credits: Hirofumi Tomita

The surgical procedure employed was based on previously reported techniques for a transumbilical laparotomy in neonates and young infants [10]. In short, a vertical, spindle-shaped incision was made around the umbilical mucosal polyp (Fig. 1A). The polyp, a minimum of the surrounding skin, and the umbilical scar (umbilical cord remnant) were released from the bilateral skin flaps with traction sutures. Then, a vertical incision was made cranially in the linea alba toward the umbilical scar to access the preperitoneal space and extended caudally around the umbilical scar (Fig. 1B). The peritoneum was incised, and the abdominal cavity was explored for any lesions continuous with the umbilical scar (Fig. 1C). To prevent postoperative adhesion, no exploratory surgery was performed for other intestinal anomalies in the absence of any lesion that was continuous with the umbilicus. The umbilical scar in each case was totally resected along with the dorsal peritoneum after making a caudal incision between the scar and the linea alba (Fig. 1D). Subsequently, the fascia was closed longitudinally with 2-0 multifilament continuous absorbable sutures (Fig. 1E), and the halves of the skin flaps were longitudinally sutured together using dermal 5-0 absorbable multifilament sutures while anchoring the bottom of the umbilicus to the fascia (Fig. 1F).

## Results

**Figure 2:**
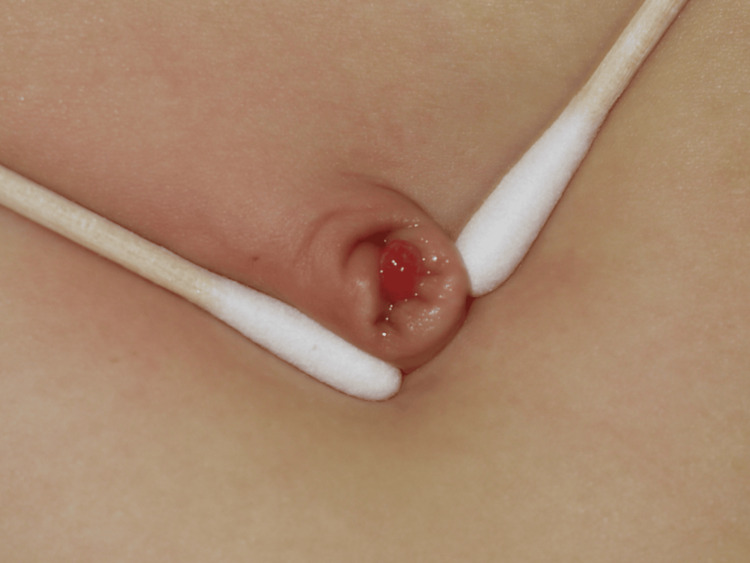
Typical appearance of the umbilical polyp The characteristic smooth surface and bright red color can be seen. The patient was female, aged three years.

**Figure 3:**
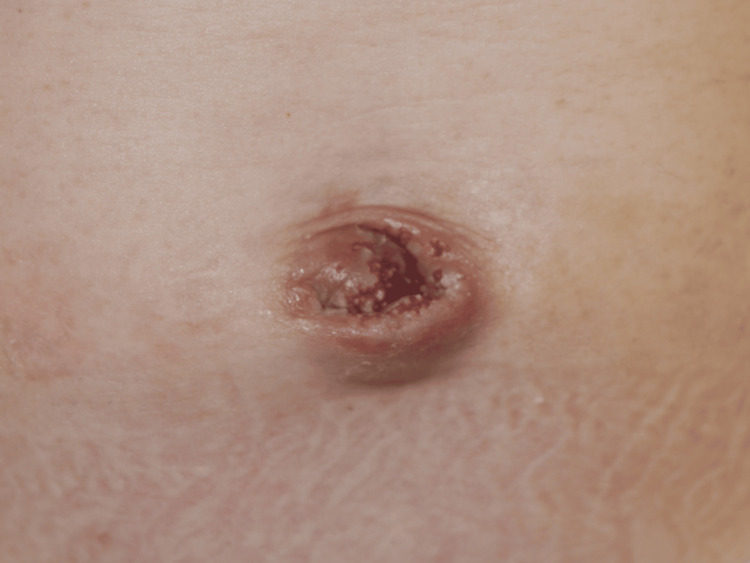
Intractable skin erosion after an umbilical polyp ligation in a male patient aged seven months Surgery and histopathological analysis confirmed the presence of an umbilical sinus involving the gastric mucosa deep within the umbilicus.

Fourteen patients with an umbilical mucosal polyp were enrolled (summarized in Table 1). Fig. 2 shows the typical appearance of the lesion. Eight (57.1%) patients were male, the median age at the first visit was 10 months (range: one month to seven years), and the median duration of illness was seven months (range: one month to seven years). Symptoms included effusion, bleeding, and foul-smelling discharge in 12 (85.7%), 10 (71.4%), and three (21.4%) patients, respectively. The patients’ previous treatment history included silver nitrate cautery, ligation, and topical steroids in eight (57.1%), four (28.6%), and 10 (71.4%) patients, respectively. An intractable skin erosion developed after ligation in two (14.3%) patients (Fig. 3). No other relationship was found between the symptoms and the past treatment history.

**Table 1:** Characteristics and treatments in 14 patients with an umbilical mucosal polyp

Male sex	8 (57.1%)
Age at initial referral	10 months (1 month–7 years)
Period of illness	7 months (1 month–7 years)
Symptoms	
Effusion	12 (85.7%)
Bleeding	10 (71.4%)
Foul-smelling discharge	3 (21.4%)
Intractable skin erosion	2 (14.3%)
Previous treatment	
Silver nitrate cautery	8 (57.1%)
Ligation	4 (28.6%)
Topical steroid	10 (71.4%)
Our treatment	
Silver nitrate cautery	4 (28.6%)
Ligation	1 (7.1%)
Topical steroid	3 (21.4%)
Surgery	13 (92.9%)

**Figure 4:**
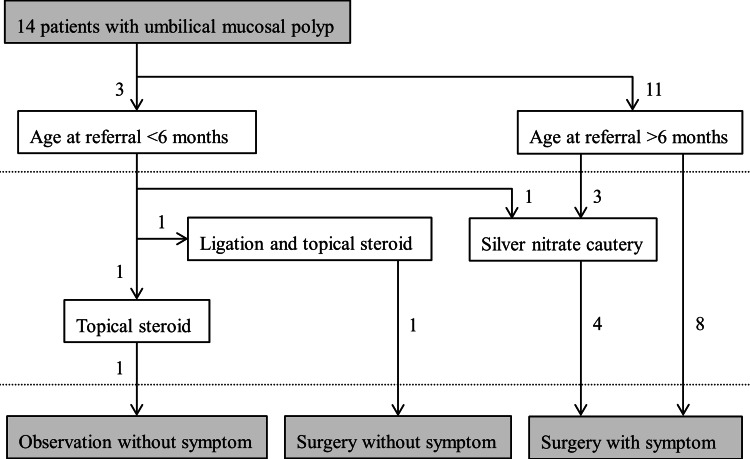
Flow chart of patient management

Fig. 4 shows a flow chart of the patients’ management. Three patients were younger than six months; one of these improved with topical steroid application after the initial visit at the age of four months and declined surgery; one improved following ligation and topical steroid application at the age of one month and underwent an exploratory laparotomy and umbilical protrusion repair at the age of nine months after histological analysis of the intestinal mucosa; finally, one failed to improve after silver nitrate cautery at the age of two months and underwent surgery at the age of 10 months. The remaining 11 patients, who were older than six months at their initial presentation, underwent surgery. Three patients failed to respond to silver nitrate cautery.

**Figure 5:**
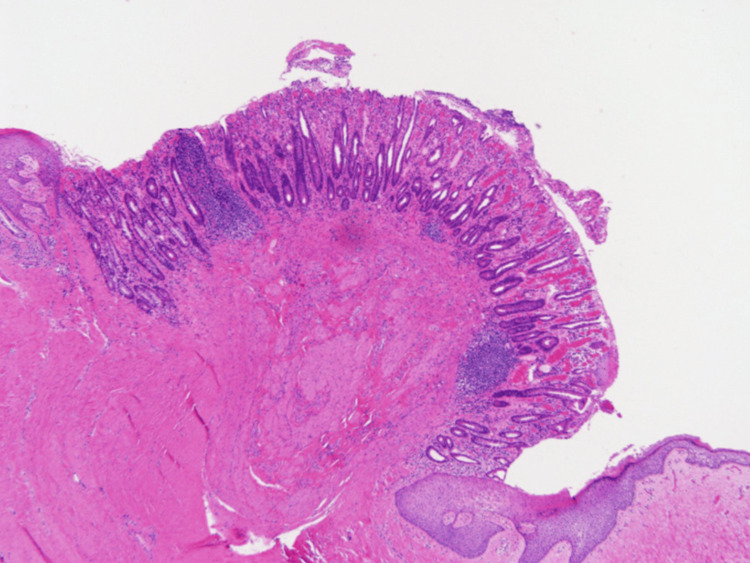
Microscopy of an umbilical mucosal polyp The presence of intestinal mucosa on a histopathological examination confirmed the diagnosis. The patient was female, aged one year.

**Figure 6:**
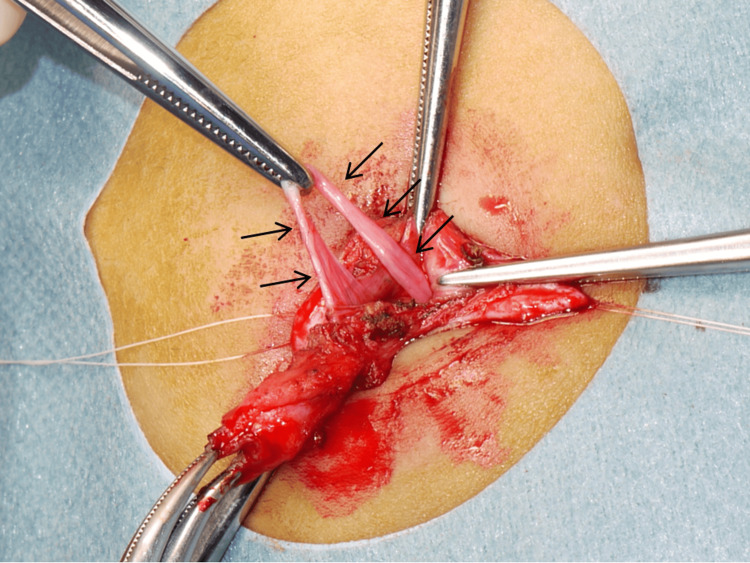
Photograph taken during an exploratory laparotomy An omphalomesenteric band (arrows) was detected in the female patient, aged five years.

**Figure 7:**
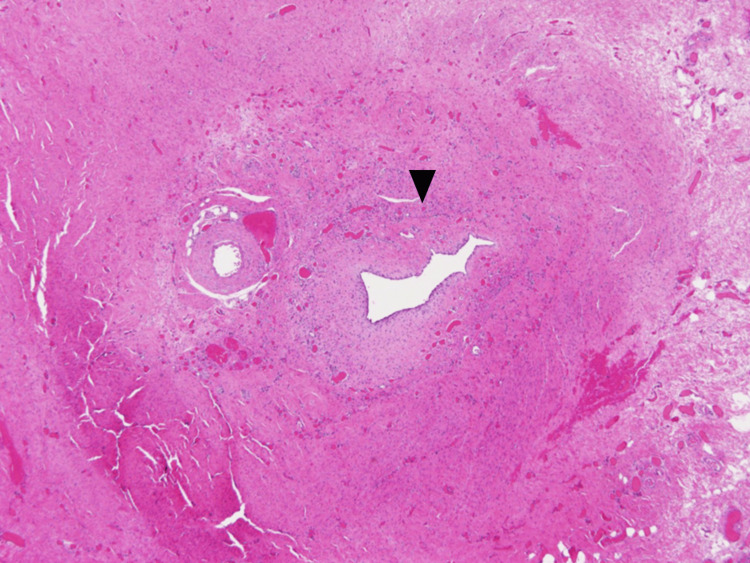
Microscopy of an umbilical scar in a female patient, aged one year An umbilical cyst with a columnar epithelial lining (arrowhead) was detected along with a vitelline vessel remnant.

**Figure 8:**
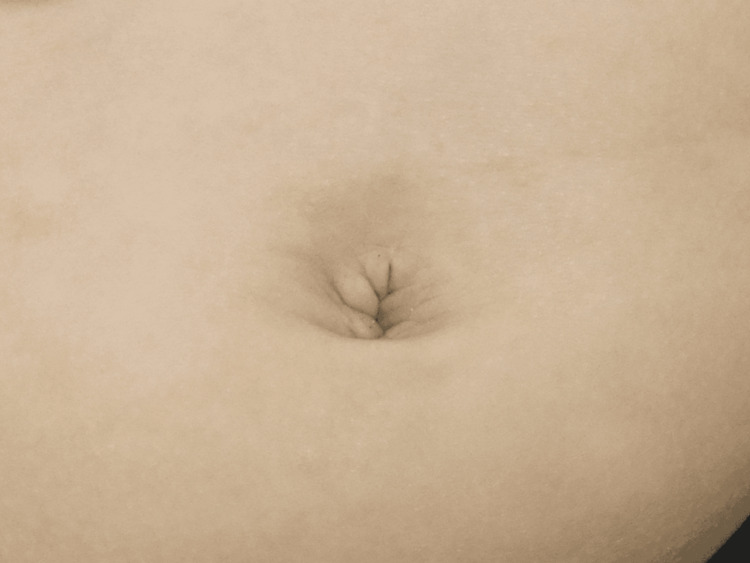
Typical postoperative appearance Most patients achieved satisfactory cosmetic results. The patient in the photograph was female, aged one year.

In total, 13 patients received surgery at the age of 15 months (nine months to seven years). The median operative time was 46 minutes (32-83 minutes), and the histological examinations revealed intestinal mucosa in all the patients (Fig. 5). Four of the 13 patients (30.8%) had a lesion deep within the umbilicus; of these, one had an omphalomesenteric band detected during intraperitoneal exploration (Fig. 6), one had a histopathologically detected umbilical cyst with columnar epithelial lining in the umbilical scar (Fig. 7), and two had intractable skin erosion after ligation of the umbilical polyp indicating an umbilical sinus involving the gastric mucosa (Fig. 3). One of the last two patients experienced temporary, postoperative granulation followed by hypertrophic scar formation as a complication. The remaining 12 patients had an uneventful postoperative course with satisfactory cosmetic results (Fig. 8).

## Discussion

Although reports of umbilical mucosal polyps are relatively rare, Matsukawa et al. described 13 patients with a pathologically confirmed (n = 10) or clinically suspected (n = 3) umbilical mucosal polyp among 78 patients with refractory umbilical granulation and suggested that this type of lesion was common in infancy [1]. During the study period, 15 patients received a diagnosis of umbilical granuloma (data not shown). The patients with prolonged illness tended to be referred to our department, resulting in a selection bias. Therefore, the ratio of patients with an umbilical polyp to those with an umbilical granuloma appeared less meaningful in the present study. The present report included two patients aged <6 months whose symptoms resolved without surgery, suggesting that some cases of umbilical mucosal polyp might be misdiagnosed as an umbilical granuloma. To prevent the prolongation of illness, pediatricians should become familiar with the pathognomonic appearance of umbilical mucosal polyps. More recently, Kim et al. [11] have reported the utility of ultrasound in differentiating umbilical polyps from granulomas. Although the current study was unable to apply ultrasound findings in diagnosing umbilical mucosal polyps, this imaging modality may prove useful in clinical practice.

The omphalomesenteric duct is a tubular structure joining the midgut of the early embryo to the yolk sac and is normally obliterated before birth. An omphalomesenteric duct remnant may develop into an umbilical mucosal polyp, umbilical sinus, umbilical cyst, obliterated internal duct, or Meckel’s diverticulum [2]. These anomalies originating in the omphalomesenteric duct remnant reportedly have a higher incidence in male individuals [12]. Although umbilical mucosal polyps may be associated with potentially life-threatening, internal complications, such as intestinal strangulation [2-4], it is unclear whether intraperitoneal exploration is needed in patients with an umbilical mucosal polyp. In 1979, Kutin et al. reviewed 118 patients with an umbilical polyp, 56% of whom had an associated lesion, and recommended that either a mini-laparotomy be performed or lifelong follow-up be provided along with educating the patients’ family about possible complications [3]. However, most previous cases of umbilical mucosal polyps were mentioned in case reports [4,13-18], thus raising the possibility of a publication bias.

The prevalence of intraperitoneal lesions, including umbilical mucosal polyps, therefore remains unclear. Few reports have investigated the prevalence of intraperitoneal lesions on the basis of exploratory surgical findings in patients with an umbilical mucosal polyp. Steck et al. described ten patients with an internal lesion involving an umbilical mucosal polyp and a patent omphalomesenteric duct among 16 patients with a cutaneous omphalomesenteric duct remnant [2]. Kutin et al. described two patients with Meckel’s diverticulum or an obliterated omphalomesenteric duct among six patients with an umbilical mucosal polyp [3]. Pacilli et al. and Matsukawa et al. found no other types of lesions in seven and six patients, respectively, with an umbilical mucosal polyp [1,19]. Moreover, the seven patients in the study by Pacilli et al., who were followed without abdominal exploration, were asymptomatic at a median period of 5.8 years (range: 0.9-13.7 years), suggesting that abdominal exploration may be unnecessary in some patients with an umbilical mucosal polyp [19].

Previous studies have generally provided few details of the procedure for excising an umbilical mucosal polyp or performing exploratory laparotomy [2,3,13-17,19]. Reports by dermatologists have described a shave biopsy or shave excision as a possible method [13,18]. One of the patients in these studies underwent a shave biopsy and then cauterization of the base of the excised umbilical polyp, indicating that the excision was only superficial [13]. By contrast, Miyagi et al. resected the tissue beneath the umbilicus containing peritoneal tissue [4] on the assumption that it was continuous with the umbilical scar, a method also endorsed by Matsukawa et al. [1]. In some of the patients in the present study as well, a hidden umbilical sinus or cyst was found within the umbilical scar. Thus, the umbilical scarectomy is recommended to prevent the recurrence of umbilical polyps.

The procedure for conducting an exploratory laparotomy varies. Kutin et al. used a small, right-lower-quadrant incision or transverse paraumbilical incision [3]; Pacilli et al. performed a mini-laparotomy or laparoscopy [19]; Miyagi et al. performed a laparotomy via an infraumbilical incision [4]; and Hou performed a transumbilical mini-laparotomy [17]. Weighing the benefits and disadvantages of surgery is of paramount importance. The scarectomy performed in the present study involved a transumbilical approach via a wound created by the removal of the umbilical polyp together with the umbilical scar. This procedure minimizes the operative time and scarring. Thus, the benefits of an exploratory laparotomy, such as preventing strangulation caused by the rare occurrence of an omphalomesenteric band, outweigh the potential harms posed by the invasiveness of surgery.

The present study has some limitations. First, the diagnosis of umbilical mucosal polyp was not confirmed in one of the patients because the intestinal mucosa was not histopathologically analyzed. However, given that the remaining 13 patients had histopathological evidence of intestinal mucosa as expected at the time of enrollment, the diagnosis of umbilical mucosal polyp by an experienced surgeon based on the patient’s clinical history and the characteristic appearance of the lesion was likely accurate. Second, in the absence of a lesion that was continuous with the umbilicus, no exploratory surgery was performed for other intra-peritoneal lesions in the belief that doing so might lengthen the wound, impair cosmesis, and expose the intestines, potentially leading to adhesion and intestinal obstruction. As a result, the estimate of the prevalence of intraperitoneal lesions in the present study is uncertain. Patients’ parents or guardians should be informed about the clinical symptoms of Meckel’s diverticulum.

## Conclusions

Umbilical mucosal polyps can be diagnosed on the basis of their clinical history and characteristic appearance. To prevent the prolongation of illness, pediatricians should become familiar with the pathognomonic features of the umbilical mucosal polyp: a smooth surface, bright red coloration, ineffectiveness of non-surgical interventions, and, occasionally, dermatitis of the surrounding skin caused by digestive discharge. Because patients with an umbilical polyp may have a hidden umbilical sinus or cyst within the umbilical scar, an umbilical scarectomy during surgery for umbilical mucosal polyp removal is recommended to prevent a recurrence. Exploratory laparotomy via the wound created by an umbilical scarectomy minimizes the operative time and scarring. Thus, in patients with an umbilical mucosal polyp, the benefits of an exploratory laparotomy, such as preventing strangulation caused by the rare occurrence of an omphalomesenteric band, outweigh the potential harms posed by the invasiveness of the surgery.

## References

[1] Matsukawa Y, Yoshitoshi EY, Wakasa T (2010). Clinical features and differential diagnosis of umbilical polyp and umbilical granuloma [in Japanese]. Jpn J Pediatr Surg.

[2] Steck WD, Helwig EB (1964). Cutaneous remnants of the omphalomesenteric duct. Arch Dermatol.

[3] Kutin N, Allen J, Jewett T (1979). The umbilical polyp. J Pediatr Surg.

[4] Miyagi H, Honda S, Minato M, Okada T, Hatanaka KC, Taketomi A (2016). Impact of umbilical polyp resection: a report and literature review. Afr J Paediatr Surg.

[5] Hegazy AA (2016). Anatomy and embryology of umbilicus in newborns: a review and clinical correlations. Front Med.

[6] Nakamura Y, Teramoto Y, Tanaka R (2014). Surgical management of umbilical basal cell carcinoma: published work review and the optimal depth of surgical excision. J Dermatol.

[7] Bongini M, Tanini S, Messineo A, Facchini F, Ghionzoli M (2015). Umbilical reconstruction in children: a simplified operative technique. Aesthetic Plast Surg.

[8] Cezarino BN, Lopes RI, Berjeaut RH, Dénes FT (2021). Laparoscopic hidden incision endoscopic surgery (hides) nephrectomy vs. traditional laparoscopic nephrectomy: non-inferior surgical outcomes and better cosmetic results. J Pediatr Urol.

[9] Elias WJ, Simmons NE, Kaptain GJ, Chadduck JB, Whitehill R (2000). Complications of posterior lumbar interbody fusion when using a titanium threaded cage device. J Neurosurg.

[10] Tomita H, Shimojima N, Shimotakahara A (2023). Vertical umbilical incision achieves better cosmesis than periumbilical incision in neonates and infants. Cureus.

[11] Kim DH, Lee HJ, Kim JY, Jung HR (2020). Differential diagnosis of umbilical polyps and granulomas in children: sonographic and pathologic correlations. Ultrasonography.

[12] Kadian YS, Verma A, Rattan KN, Kajal P (2016). Vitellointestinal duct anomalies in infancy. J Neonatal Surg.

[13] Swanson DL, Pakzad B (2005). An umbilical polyp in an infant. Cutis.

[14] You Y, Yang X, Hao F, Zhong B (2009). The umbilical polyp: a report of two cases and literature review. Int J Dermatol.

[15] Hsu JW, Tom WL (2011). Omphalomesenteric duct remnants: umbilical versus umbilical cord lesions. Pediatr Dermatol.

[16] Pérez-Mesonero R, Melgar-Molero V, Martín-Fuentes A (2017). Congenital umbilical nodule in a 1-year-old infant [Article in Portuguese]. Actas Dermo-Sifiliográficas (English Edition).

[17] Hou YL, Lin JY (2019). Surgical abdominal exploration in children with umbilical ectopic gastrointestinal tissue. J Pediatr Surg Case Rep.

[18] Kang BY, Mitchell DC, Stepenaskie S, Smidt AC (2023). Umbilical nodule present since birth. Pediatr Dermatol.

[19] Pacilli M, Sebire NJ, Maritsi D, Kiely EM, Drake DP, Curry JI, Pierro A (2007). Umbilical polyp in infants and children. Eur J Pediatr Surg.

